# Creatine supplementation in soccer players: a systematic review

**DOI:** 10.1080/15502783.2026.2735569

**Published:** 2026-10-02

**Authors:** Damoon Ashtary-Larky, Darren G. Candow, Jose Antonio, Christian Quiceno, Fabrizio Angelini, Scott C. Forbes, Sajjad Moradi, Fatemeh Kazeminasab, Reza Bagheri

**Affiliations:** a Nutrition and Metabolic Diseases Research Center, Ahvaz Jundishapur University of Medical Sciences, Ahvaz, Iran; b Faculty of Kinesiology and Health Studies, University of Regina, Regina, Canada; c Department of Health and Human Performance, Nova Southeastern University, Davie, FL, USA; d Director of Sports Medicine department, Inter Bogotá, Colombia; e Society of Sport Nutrition and Wellness, Pisa, Italy; f Department of Physical Education Studies, Brandon University, Brandon, MB, Canada; g Department of Nutrition and Food Sciences, Maragheh University of Medical Sciences, Maragheh, Iran; h Department of Physical Education and Sports Science, Faculty of Humanities, University of Kashan, Kashan, Iran; i Department of Exercise Physiology, University of Isfahan, Isfahan, Iran

**Keywords:** Football, repeated-sprint performance, anaerobic power, muscle strength, body composition, recovery, fatigue, team sports, ergogenic aids

## Abstract

**Aim:**

Soccer places repeated demands on short-duration, high-intensity performance, including sprints, accelerations, jumps, and technical actions performed over the course of a match. Because these efforts depend heavily on rapid adenosine triphosphate (ATP) resynthesis, creatine is a plausible ergogenic aid for soccer players. However, findings in soccer-specific studies have been inconsistent, and previous reviews have addressed narrower questions using smaller evidence sets. This systematic review therefore examined the effects of creatine supplementation on performance, body composition, and recovery/fatigue-related outcomes in soccer players.

**Methods:**

PubMed, Scopus, and Web of Science were searched from inception to 15 May 2025 in accordance with the Preferred Reporting Items for Systematic Reviews and Meta-Analyses (PRISMA) 2020 statement. We included controlled clinical trials of creatine supplementation in male or female soccer players of any age or competitive level when an eligible placebo, control, or comparison condition was available. Outcomes were grouped into performance, body composition, and recovery/fatigue domains. Domains from the revised Cochrane risk-of-bias tool for randomised trials (RoB 2) and the Risk Of Bias In Non-randomised Studies - of Interventions (ROBINS-I) tool were used to produce study-level methodological summaries rather than formal result-specific risk-of-bias assessments. Because the studies differed substantially in design, supplementation protocols, populations, and outcomes, findings were synthesised qualitatively rather than pooled in a meta-analysis.

**Results:**

Source-level verification left 45 eligible reports representing 41 unique studies: 28 randomised parallel-group studies, 2 randomised crossover/repeated-condition studies, and 11 non-randomised or allocation-unverifiable controlled studies. Some trials reported favourable findings for repeated-sprint performance, anaerobic power, and muscular strength, but the comparative evidence was inconsistent and the certainty of evidence was very low. Findings for vertical jump performance and technical skills were mixed, while change-of-direction and endurance outcomes showed no consistent benefit. Recovery- and fatigue-related findings were also largely null or inconsistent. Body mass often increased, particularly after loading protocols, whereas evidence for gains in lean mass was limited and inconsistent.

**Conclusion:**

The soccer-specific evidence for creatine supplementation remains suggestive rather than conclusive. Favourable findings have been reported for some repeated-sprint, anaerobic-power, and strength outcomes, but the comparative evidence is heterogeneous, often limited by unclear or nonsignificant between-group effects, and of very low certainty. Firm conclusions about efficacy are therefore not possible. Body mass commonly increased, especially after loading protocols, while effects on lean mass, endurance, change-of-direction ability, and recovery/fatigue-related outcomes remain uncertain.

## 1. Introduction

Soccer is a widely played sport worldwide with over 275 million registered participants. Soccer is a physically demanding intermittent sport that combines aerobic endurance with bursts of high-intensity sprints, changes in direction, jumping, and repeated tackling or kicking. Players must maintain a high level of performance throughout the match, which lasts ~90 minutes (two 45-minute halves) and often exceeds 10 km of total running distance [1]. Importantly, match-play analysis indicates that players may perform 30-50 high-intensity sprints per match, reach peak sprint speeds exceeding 30 km/h, and execute numerous explosive accelerations, decelerations, and jumps, particularly during decisive phases of play [2]. Both a structured training programme and a proper diet are foundational to obtaining these performance characteristics [3,4]. Furthermore, supplements are purported to reduce fatigue, enhance recovery, and improve performance and body composition [5–8].

Creatine is a naturally occurring nitrogen-containing compound (an amino acid-derived molecule) and is among the most extensively researched and commonly used ergogenic aids in both professional and recreational athletes. Creatine plays an important role in energy metabolism by rapidly replenishing adenosine triphosphate (ATP) through the phosphocreatine system during short-duration, high-intensity efforts [9]. Mechanistically, creatine exerts its effects via increased intramuscular phosphocreatine stores, cellular hydration, and potentially by modulating satellite cell activity and altering anabolic/catabolic signalling [10]. A large amount of literature has shown that creatine supplementation can improve maximal power output, repeated sprint ability, strength gains, lean body mass, and recovery [11,12]. These performance enhancing effects, in theory, may benefit team-sport athletes such as soccer players. Creatine supplementation has demonstrated benefits in numerous sports involving high-intensity efforts, such as sprinting, weightlifting, American football, and rugby [13]. Despite its strong theoretical foundation, findings on the efficacy of creatine supplementation in soccer players remain inconsistent. Early investigations in the late 1990s gave inconclusive results. For example, one of the first studies reported no improvement in sprint performance in soccer players after creatine loading [14]. In contrast, subsequent trials found positive effects on sprint and jump performance [15,16], while others reported limited benefits [17].

Previous systematic reviews and meta-analyses published on creatine and soccer performance have generally addressed narrower eligibility questions and included smaller evidence sets. For instance, the review conducted by Mielgo-Ayuso et al. (2019) included nine trials [18], while more recently Guevara et al. (2024) incorporated six studies [19], reflecting a narrower scope rather than necessarily a methodological deficiency. These earlier reviews primarily emphasised selected performance variables, whereas the present review also considered body composition and recovery/fatigue-related outcomes. Moreover, the heterogeneity across individual studies in terms of sample size, supplementation protocols (e.g. dosage, duration, loading vs. non-loading), participant characteristics (e.g. age, sex, training status), and outcome measures complicates the interpretation and generalisation of results. This difference in review scope explains, at least in part, why fewer studies were included previously. Across the underlying primary studies, substantial heterogeneity in samples, supplementation protocols, and outcome measures remains an important barrier to direct comparison and practical synthesis.

Taken together, the conflicting findings and the growing emphasis on evidence-based supplementation in team sports justify a soccer-specific synthesis. Accordingly, this review evaluated the effects of creatine supplementation on performance, body composition, and recovery/fatigue-related outcomes in male and female soccer players across a range of competitive levels.

## 2. Materials and methods

### 2.1. Literature search

This systematic review followed the Preferred Reporting Items for Systematic Reviews and Meta-Analyses (PRISMA) 2020 statement and was registered in the International Prospective Register of Systematic Reviews (PROSPERO) under the registration code [CRD420261324016]. PubMed, Scopus, and Web of Science were searched from database inception to 15 May 2025; the complete search strategies are provided in Supplementary Table S1. Reference lists of relevant articles and previous reviews were also checked as a supplementary verification step. Grey literature and unpublished studies were not searched systematically. Conference abstracts and congress proceedings were eligible when they provided enough methodological and outcome information for extraction. No language restrictions were applied. The review question followed the Population, Intervention, Comparison, and Outcomes (PICO) framework: soccer players (Population), creatine supplementation (Intervention), placebo/control conditions (Comparison), and performance, body composition, or recovery/fatigue outcomes (Outcomes). Because records identified through citation checking were not prospectively logged as a separate stream, they are not shown separately in Figure 1.

**Figure 1.**
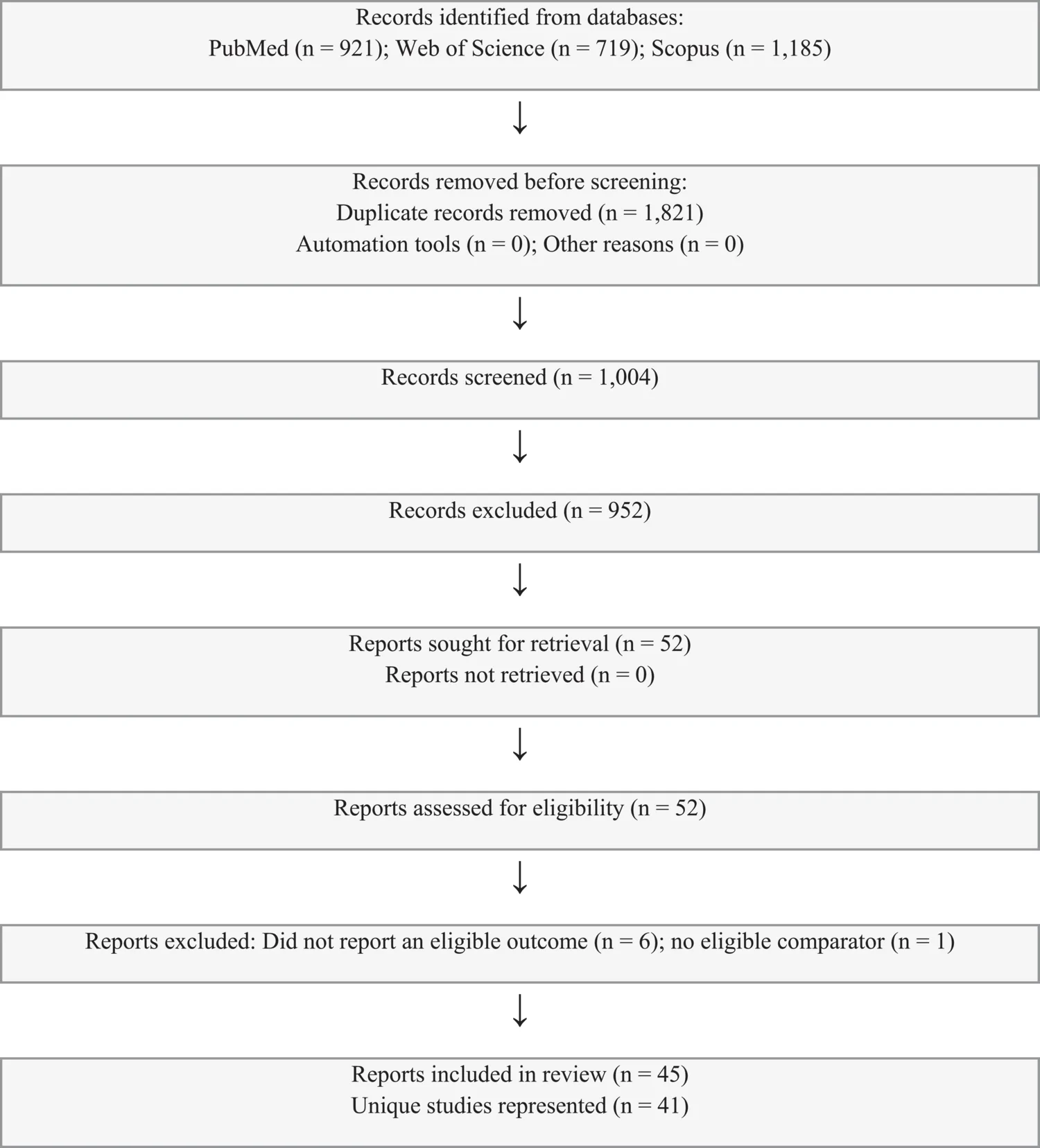
Flow diagram of study selection for the systematic review. Note: Reference-list checking was used as a supplementary verification step, but yields were not prospectively logged as a separate identification stream; the diagram therefore presents the database-search pathway and report-level eligibility counts.

### 2.2. Eligibility criteria

Studies were included if they met the following criteria:

1. randomised or non-randomised controlled clinical trials;
2. conducted on male or female soccer players of any age or competitive level;
3. investigated creatine supplementation as the primary intervention, regardless of dosage or duration;
4. included a placebo, control, or comparison condition; and
5. reported at least one outcome related to performance (e.g. sprint, power, jump performance), recovery/fatigue (e.g. markers of muscle damage, fatigue index, lactate), or body composition (e.g. fat mass, lean mass, body weight).

Conference abstracts and congress proceedings were considered eligible only when sufficient methodological and outcome data were available for extraction.

### 2.3. Exclusion criteria

Studies were excluded if they:

1. used observational designs, case reports, review articles, editorials, or commentaries;
2. did not involve soccer players;
3. investigated multi-ingredient supplements in which the independent effects of creatine could not be isolated; or
4. did not provide sufficient outcome data for extraction.

Participant training status and training protocols were not restricted, provided that training conditions were reasonably comparable between intervention and control groups within each study.

### 2.4. Study selection (screening process)

All database records were imported into reference-management software and deduplicated. Two reviewers (DAL and RB) independently screened titles and abstracts and then assessed potentially eligible full texts. Disagreements were resolved by discussion and consensus, with a third reviewer (FK) available when needed. Reports arising from the same study population were reviewed together and treated as a single study to avoid double-counting. Inter-rater agreement was not quantified statistically (for example, with Cohen's kappa).

### 2.5. Data extraction

Two reviewers (DAL and RB) independently extracted study characteristics using a standardised form. Extracted information included author and publication year, country, participant characteristics (age, sex, training status or competitive level, and sample size), study design, creatine form, dose and duration, outcomes, and the main findings within each outcome domain. When outcome data were missing or incompletely reported, we used the information available in the original report and did not impute missing values. Multiple reports from the same study population were reviewed together to avoid duplicate data. Disagreements were resolved through discussion and consensus, with third-reviewer arbitration when required. Study authors were not contacted for additional data.

### 2.6. Methodological risk-of-bias assessment

Risk-of-bias domains were examined with design-appropriate tools and summarised at the level of the unique study. The 28 randomised parallel-group studies were appraised using domains from the revised Cochrane risk-of-bias tool for randomised trials (RoB 2); the 2 randomised crossover/repeated-condition studies were considered using crossover-specific RoB 2 domains, including period and carryover effects; and the 11 non-randomised or allocation-unverifiable controlled studies were considered using the original seven-domain Risk Of Bias In Non-randomised Studies - of Interventions (ROBINS-I) framework. The ROBINS-I group included studies with explicitly non-random allocation, matched or fixed-sequence allocation without demonstrated randomisation, and older or abstract-only reports in which random allocation could not be verified. Reports from the same parent cohort were summarised once. The original review workflow produced one methodological profile per study rather than a separate formal risk-of-bias assessment for each individual outcome or result. Because RoB 2 and ROBINS-I are intended for result-specific assessment, Figures 2 and 3 should be read as study-level methodological summaries. We therefore do not assume that risk of bias was identical for every outcome reported within a study.

**Figure 2.**
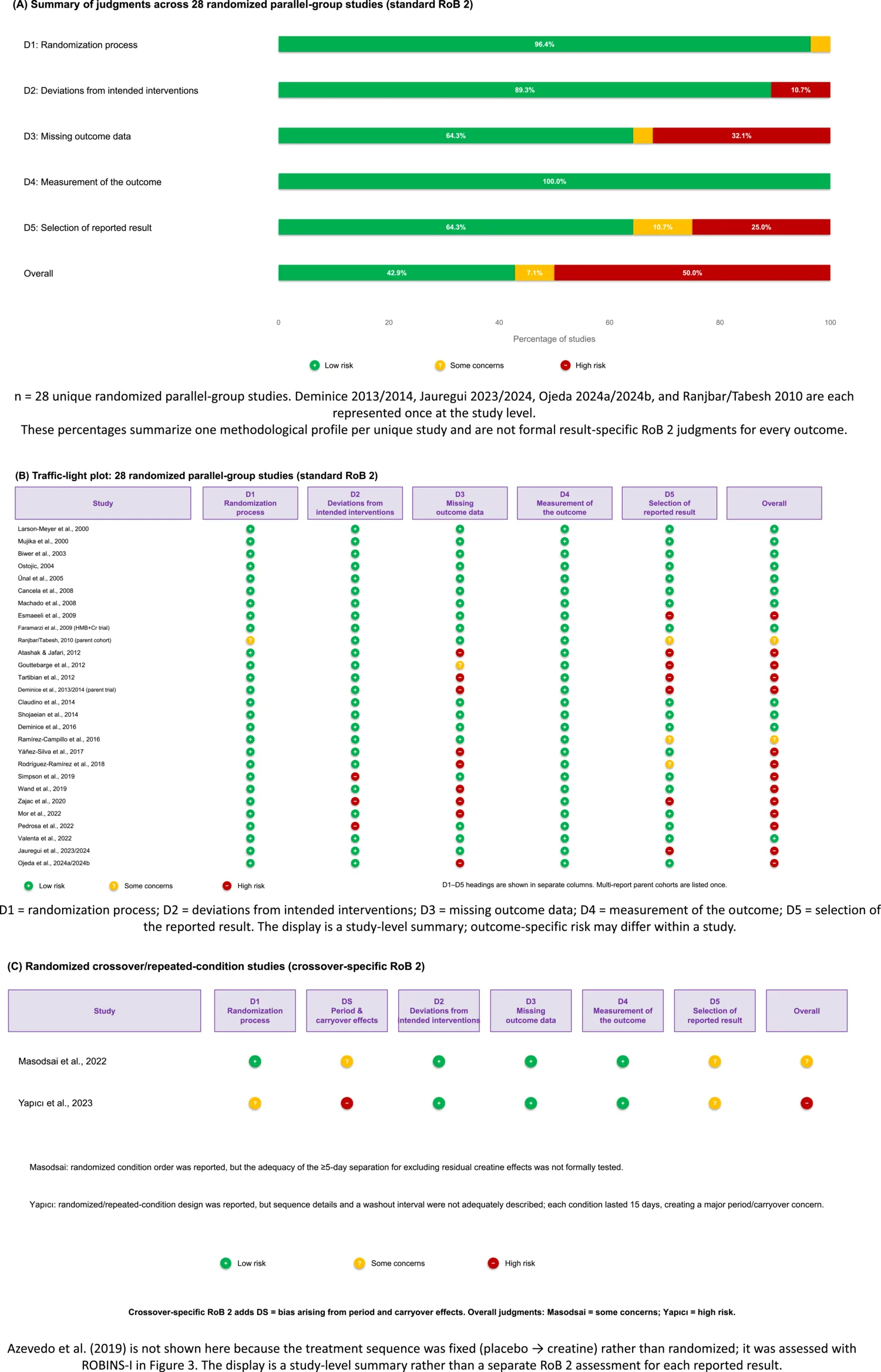
Study-level methodological risk-of-bias summary using design-specific RoB 2 domains.

**Figure 3.**
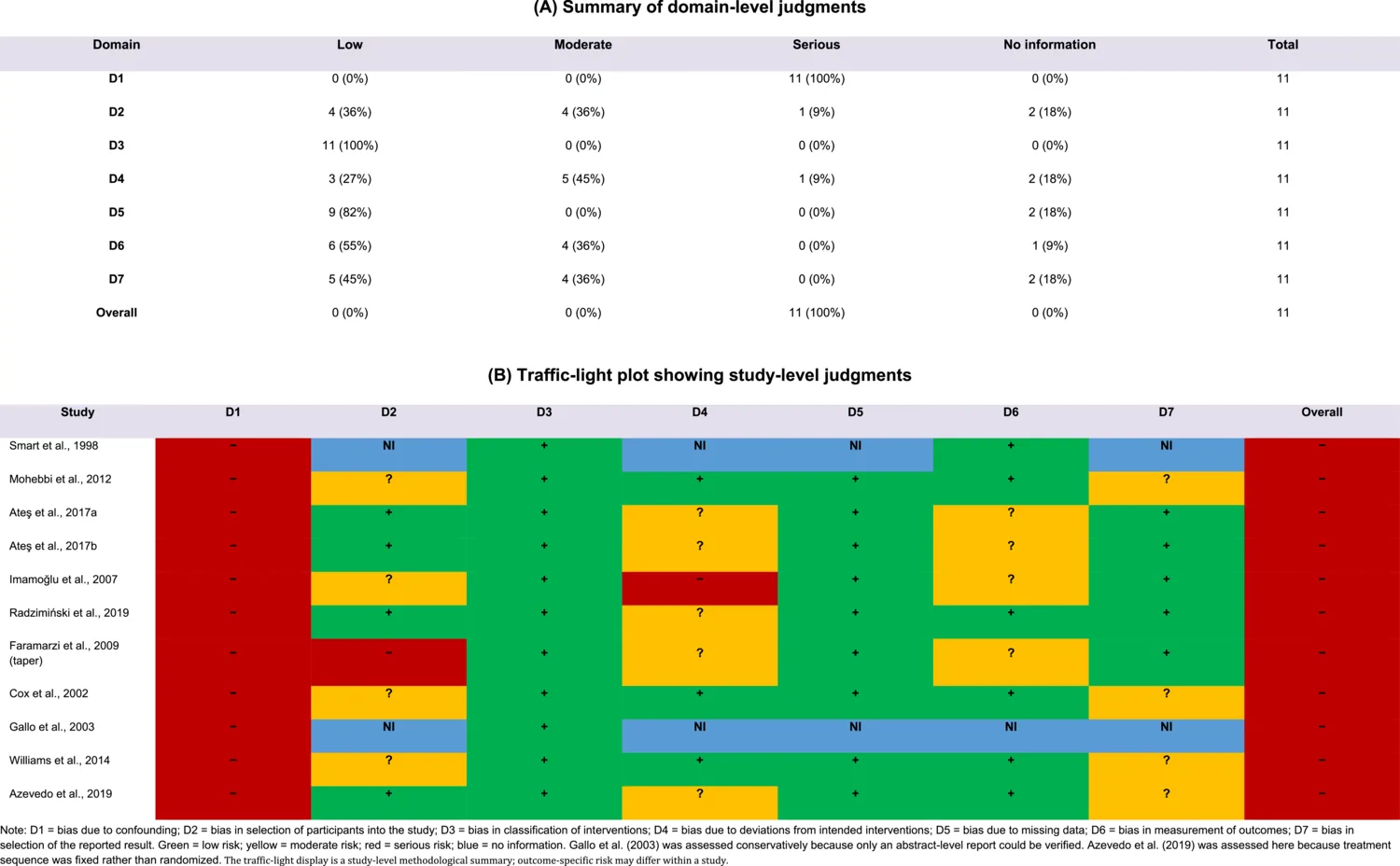
Study-level methodological summary using ROBINS-I domains for 11 non-randomised or allocation-unverifiable controlled studies. Note: D1 = bias due to confounding; D2 = bias in selection of participants into the study; D3 = bias in classification of interventions; D4 = bias due to deviations from intended interventions; D5 = bias due to missing data; D6 = bias in measurement of outcomes; D7 = bias in selection of the reported result. Green = low risk; yellow = moderate risk; red = serious risk; blue = no information. Gallo et al. [20] was assessed conservatively because only an abstract-level report could be verified. Azevedo et al. [21] was assessed here because treatment sequence was fixed rather than randomised. The traffic-light display is a study-level methodological summary; outcome-specific risk may differ within a study.

### 2.7. Certainty of evidence assessment

The certainty of evidence for the main outcome domains was assessed using the Grading of Recommendations Assessment, Development and Evaluation (GRADE) approach [22]. Evidence was evaluated across methodological limitations, inconsistency, imprecision, indirectness, and publication bias. Because each broad domain contained a mixture of randomised and non-randomised evidence, certainty judgements were anchored to the randomised evidence stream; non-randomised studies were treated as supportive/contextual evidence and did not increase certainty. Publication bias was rated as suspected because the search used three databases with outcome-constrained syntax, grey literature was not systematically searched, several reports were small or abstract-only, and selective-reporting concerns were common. Imprecision was considered serious when the evidence was dominated by small studies and no precise pooled estimate was available. These are domain-level summary judgements rather than outcome-specific GRADE ratings, an important limitation given the heterogeneity within the broad performance and recovery/fatigue domains.

### 2.8. Synthesis of results

A meta-analysis was not undertaken because the studies differed substantially in creatine dose and duration, participant characteristics, and outcome measurement. We therefore used a structured qualitative synthesis. For each eligible comparison, a finding was classified as favourable comparative evidence when a statistically supported between-group difference or group-by-time interaction favoured creatine; as null comparative evidence when the between-group contrast or interaction was explicitly nonsignificant; or as within-group-only/statistically unclear evidence when only within-group significance was reported or an interpretable between-group statistic could not be established. Timing-only and formulation-only comparisons were retained descriptively but were not treated as evidence of creatine efficacy versus a control condition. Study size and risk of bias were not assigned numerical weights, although greater interpretive emphasis was placed on valid comparative findings from randomised studies and high-risk or non-randomised evidence was treated cautiously.

### 2.9. Synthesis without meta-analysis (SWiM)

Due to substantial heterogeneity in supplementation protocols, intervention durations, participant characteristics, and outcome assessments, a quantitative meta-analysis was not considered appropriate. Findings were synthesised narratively in accordance with the SWiM reporting guidance. Studies were grouped into body composition, performance, and recovery/fatigue domains. The synthesis distinguished statistically supported between-group/group-by-time effects from explicit between-group null findings and from within-group-only or statistically unclear findings. Timing/formulation-only comparisons were not counted as creatine-versus-control efficacy evidence. No inferential vote-counting test or numerical weighting by sample size was performed; qualitative conclusions emphasised the consistency, comparative validity, and methodological credibility of the contributing evidence [23].

## 3. Results

### 3.1. Overview of included studies

The source-level verification set comprised the reports cited below; after excluding one conference abstract that did not describe an eligible comparator, 45 eligible reports representing 41 unique studies remained (Figure 1) [14–17,20,21,24–63]. The main characteristics and findings of the included studies, including participant details, supplementation protocols, and assessed outcomes, are summarised in Table 1. These studies were conducted across countries, with the majority based in Brazil, Spain, and Iran. The sample populations consisted primarily of male athletes, though a small number of studies also included female soccer players. Participant age ranged from adolescent players to professional adult athletes, covering a wide range of performance levels from amateur to elite.

**Table 1.** Characteristics and main findings of studies investigating the effects of creatine supplementation on body composition, performance, and recovery/fatigue-related outcomes in soccer players.

Study (Author, Year)	Participants	Design/Duration	Creatine Protocol	Outcomes Measured	Main Findings
**Smart et al., 1998**	11 Bruneian national squad soccer players	NR, DB, PC; matched/assigned without reported randomisation (ROBINS-I)	Cr 24 g/day for 6 days	Plasma concentrations of lactate, uric acid and hypoxanthine, sprint performance, body mass	**Body Composition:** Body mass significantly increased in the Cr group. **Performance:** There were no significant changes in sprint performance between the two testing sessions for either group. **Recovery/Fatigue:** Plasma concentrations of lactic acid, uric acid, and hypoxanthine at baseline and post-exercise showed no significant differences between groups.
**Larson-Meyer et al., 2000**	14 female collegiate soccer players	R, DB, PC	Cr monohydrate:15 g/day for 1 week, then ~5 g/day for 12 weeks	maximal strength, anaerobic running performance and body composition (DEXA)	**Body Composition:** Both groups increased FFM and body mass with training; however, Cr did not produce additional gains beyond training alone. FM remained unchanged following the interventions. **Performance:** Cr supplementation led to greater improvements in bench press (0–5 wks) and full squat strength (5–13 wks) compared to placebo. **Recovery/Fatigue:** Not measured.
**Mujika et al., 2000**	17 highly trained male soccer players	R, DB, PC	5 g of Cr, 4 times per day for 6 days	CMJT (three maximal vertical jumps), RST (six maximal 15-m sprints with 30 s rest), IET (forty 15-s bouts of high-intensity exercise and thirty-nine 10-s low intensity exercise), recovery CMJT (a maximal counter-movement jump, followed by two more jumps), blood ammonia, lactate, and urinary creatinine, body mass.	**Body Composition:** Body mass increased within the Cr group; a significant between-group treatment effect was not clearly established. **Performance:** Repeated-sprint time and vertical-jump performance improved within the Cr group but not placebo; however, a significant between-group treatment effect was not clearly established. CMJT and IET showed no detectable benefit. **Recovery/Fatigue:** The Cr group showed less fatigue over successive sprints; however, a significant between-group treatment effect was not clearly established. Post- RST and post- IET lactate, and post-RST blood ammonia, and urinary Cr did not significantly change in Cr group compared to placebo.
**Cox et al., 2002**	12 female elite soccer players	DB, PC; allocation method not verifiable as randomised (ROBINS-I)	Cr monohydrate 20 g/day for 6 days	RST, agility runs, precision ball-kicking, HR, blood lactate and pH, RPE	**Body Composition:** Body mass increased within the creatine group but not placebo; a significant between-group treatment effect was not clearly established. **Performance:** Favourable differences were reported in selected sprint and preplanned change-of-direction blocks, but average performance across the protocol did not differ significantly between conditions. Ball-kicking accuracy remained unchanged. **Recovery/Fatigue:** Cr significantly reduced post-exercise HR and blood lactate in some blocks, slightly increased blood pH in early blocks, and had no effect on RPE.
**Biwer et al., 2003**	15 college soccer players	R, DB, PC	Cr monohydrate 0.3 g/kg/day for 6 days	IET on treadmill, body mass, RPE, blood lactate	**Body Composition:** Body mass increased within the creatine condition in male participants but not female participants; the comparative treatment effect was not clearly reported. **Performance:** No significant differences were found between Cr and placebo for TTE **Recovery/Fatigue:** No significant differences were found for RPE, or blood lactate levels. Increases in RPE and lactate were due to exercise progression, not treatment effects.
**Gallo et al., 2003**	86 young male soccer players	Controlled intervention; allocation/blinding details not verifiable from available abstract (ROBINS-I)	20 g/day of Cr monohydrate for 2 weeks, followed by 5 g/day for the next 26 weeks	calf CSA; body composition	**Body Composition:** Increases in body mass, muscle mass, and calf CSA and a reduction in FM were reported; however, the full methodological report was not available and the comparative treatment effect could not be verified. Performance: Not measured. **Recovery/Fatigue:** Long-term Cr use was reported not to be associated with higher injury rates or cramping; this abstract-only evidence was interpreted cautiously.
**Ostojic, 2004**	20 young male soccer players	R, PC	Cr monohydrate 3 × 10 g/day for 7 days	soccer-specific skill tests: dribble test, sprint-power test, endurance test, and vertical jump test	**Body Composition:** Not measured. **Performance:** Post-supplementation dribble time, sprint-power performance, and vertical-jump height were significantly better in the creatine group than placebo; endurance performance showed no between-group benefit. Recovery/Fatigue: Not measured.
**Ünal et al., 2005**	30 young male soccer players	R, DB, PC	Cr monohydrate 0.25 g/kg/day for 4 days	Standing long jump, standing vertical skip tests, anaerobic Wingate anaerobic test, blood lactate	**Body Composition:** Not measured. **Performance:** Mean power, minimum power, and total work increased within the Cr group, but a significant between-group treatment contrast was not clearly reported. Peak power, vertical jump, and long-jump performance did not improve. **Recovery/Fatigue:** FI decreased within the Cr group; however, a significant between-group treatment effect was not clearly established. Post-exercise lactate levels also showed a smaller increase compared to baseline in this group. No significant changes were observed in any variables in the placebo group.
**Imamoğlu et al., 2007**	32 male trained amateur soccer players	PC; allocation method not verifiable as randomised (ROBINS-I)	20 g/day of Cr monohydrate for 1 week, followed by 5 g/day for the next 3 weeks	vertical jumping performances, leg strength, anaerobic performance capacity and sprinting times, body composition	**Body Composition:** Increases in body mass and a reduction in FM were reported after creatine supplementation; the comparative statistical basis was not clearly described in the extracted summary. **Performance:** The creatine group showed faster repeated-sprint times and greater improvements in leg strength, anaerobic capacity, and vertical-jump performance than the comparator group; however, the allocation method was not determined. Recovery/Fatigue: Not measured.
**Cancela et al., 2008**	14 male soccer players	R, DB, PC	Cr monohydrate 15 g/day for 7 days, then 3 g/day for 7 weeks	Renal (creatinine, BUN) and hepatic (total proteins and albumin, AST, ALT, γGT) markers; body mass, CK	**Body Composition:** Body mass increased within the creatine group but not placebo; a significant between-group treatment effect was not clearly reported. **Performance:** Not measured. **Recovery/Fatigue:** No significant changes were reported in renal, hepatic, or urinary markers. Total CK increased and serum glucose decreased within the creatine condition, while uric acid remained unchanged; the comparative statistical basis was not clearly described.
**Machado et al., 2008**	31 professional soccer players	R, DB, PC	Cr monohydrate 0.6 g/kg/day for 5 days	Metabolic response to exercise: blood ammonia, urea, ergometric test (GXT) according to Bruce’s protocol	**Body Composition:** Not measured. **Performance:** No significant improvement in endurance performance (GXT duration on treadmill) with Cr supplementation compared to placebo. **Recovery/Fatigue:** A smaller rise in urea was reported after creatine supplementation, while ammonia did not change and uric acid increased post-exercise in both groups; the comparative statistical basis for the urea finding was not clearly described.
**Esmaeeli et al., 2009**	21 young male soccer players	R, DB, PC	5-day loading: Cr + carbohydrate (20 g Cr + 100 g glucose/day) vs Cr-only (20 g/day) vs placebo	Body composition (mass); CMJ; RAST (6 × 35 m sprints); FI	**Body Composition:** Body mass increased within both Cr groups but not placebo; a significant between-group treatment effect was not reported. **Performance:** Within-group, the Cr and Cr + CHO groups improved in maximum and average power on the RAST, but between-group differences were not significant. No significant change in CMJ height was seen in any group. **Recovery/Fatigue:** FI did not significantly change in any group.
**Faramarzi et al., 2009**	18 male elite soccer players	NR, controlled; purposive allocation (ROBINS-I)	Cr monohydrate 10 g/day for 10 days	Vertical jump, leg strength, 60-m dash, agility; body composition (BIA)	**Body Composition:** No significant changes in FM, FFM, or body mass in either group. **Performance:** Improvements in 60-m sprint time, leg strength, and agility were reported after Cr; however, the extracted report did not clearly establish whether these represented significant between-group effects. Explosive power (vertical jump) did not significantly change in either group. **Recovery/Fatigue:** Not measured.
**Faramarzi et al., 2009**	24 well-trained soccer players (relevant HMB + Cr vs HMB contrast: *n* = 16)	R, three-arm controlled trial	HMB 3 g/day + Cr 3 g/day vs HMB 3 g/day (plus placebo arm), for 6 days	RAST, PP, MP, FI, CK, LDH	**Body Composition:** Not measured. **Performance:** The relevant creatine-specific contrast is HMB + Cr versus HMB alone; PP and MP were reported to favour the HMB + Cr condition. The placebo arm was an additional study arm and should not be interpreted as an isolated creatine-versus-placebo comparison. **Recovery/Fatigue:** FI, CK, and LDH did not significantly change following supplementation.
**Tabesh et al., 2010**	18 University of Mazandaran soccer players (6 Cr, 6 folic acid, 6 placebo)	R, DB, PC; three-arm report from same parent cohort as Ranjbar	Cr monohydrate 0.3 g/kg/day for 5 days	Serum homocysteine	**Body Composition:** Not measured. **Performance:** Not measured. **Recovery/Fatigue:** Post-exhaustive exercise homocysteine decreased in the creatine arm. This report was treated as part of the same parent cohort as Ranjbar et al. and was counted once at the study level.
**Ranjbar et al., 2010**	12 college soccer players	R, DB, PC; two-arm subset/report from same parent cohort as Tabesh	Cr monohydrate 0.3 g/kg/day for 5 days	Body composition (skinfold thickness), plasma homocysteine	**Body Composition:** Body mass and BMI increased and BFP decreased within the creatine group; comparative treatment effects were not clearly reported. **Performance:** No significant between-group difference was reported for VO₂max. **Recovery/Fatigue:** Plasma homocysteine decreased after creatine supplementation at rest and after exhaustive exercise. This 12-player report appears to represent the creatine-versus-control subset of the same parent cohort as Tabesh et al.; the cohort was counted once at the study level.
**Atashak & Jafari, 2012**	18 young male soccer players	R, DB, PC	Cr monohydrate 0.3 g/kg/day for 7 days	Indirect muscle damage markers (serum LDH, CK, CK-MB)	**Body Composition:** Not measured. **Performance:** Not measured. **Recovery/Fatigue:** Cr group showed significantly higher increases in CK and CK-MB post-exercise compared to placebo. However, changes in LDH levels were not statistically significant.
**Gouttebarge et al., 2012**	16 male professional soccer players	R, DB, PC	Cr monohydrate 20 g/day for 5 days	Body mass; vertical jump peak power	**Body Composition:** Body mass increased within the Cr group; a significant between-group treatment effect was not clearly reported. **Performance:** Vertical-jump peak power increased within the Cr group and decreased within placebo; however, a significant between-group treatment effect was not clearly reported. **Recovery/Fatigue:** Not measured.
**Mohebbi et al., 2012**	17 young soccer players	NR, DB, PC; matched groups	5 g of Cr, 4 times per day for 7 days	Speed/Agility (6 × 15 m sprint test). Skill performance (dribbling and shooting accuracy test), body composition (skinfold thickness).	**Body Composition:** Body mass and lean body mass increased within the Cr group, but a significant between-group effect was not clearly documented; BFP did not change significantly. **Performance:** Repeated-sprint time and dribbling performance improved within the Cr group, but the comparative treatment effect was not clearly documented; shooting accuracy did not change significantly. **Recovery/Fatigue:** Not measured.
**Tartibian et al., 2012**	20 young soccer players	R, DB, PC	Cr monohydrate 0.3 g/kg/day for 7 days	RST (20 m, 40 m, 60 m, 100 m, repeated 5-s sprints with 10-s rest) and knee extension strength	**Body Composition:** Not measured. **Performance:** No detectable between-group benefit was found for 20-, 40-, 60-, or 100-m sprint times or 1RM knee extension. Within-group improvements in the Cr condition do not establish a supplementation effect. **Recovery/Fatigue:** Not measured.
**Deminice et al., 2013**	25 male well-trained soccer players	R, DB, PC; same parent trial as Deminice 2014	Cr 0.3 g/kg/day for 7 days	RAST, maximal oxygen consumption, body composition, blood test	**Body Composition:** No significant changes in body mass and body fat. **Performance:** Average, maximum, and minimum power values were reported to be greater in the creatine group than placebo; the extracted summary did not specify whether these findings reflected a significant group-by-time interaction. No significant difference in maximal oxygen consumption was observed between groups. **Recovery/Fatigue:** FI did not differ between groups, and no effect was reported for post-exercise blood lactate or oxidative-stress markers. Smaller inflammatory-marker responses were reported in the creatine group. LDH increased within placebo but not creatine, while antioxidant enzyme activity increased similarly in both groups; the comparative basis of the LDH finding was not clearly described.
**Deminice et al., 2014**	23 young soccer players	R, DB, PC; 23-player outcome subset of same parent trial as Deminice 2013	Cr 0.3 g/kg/day for 7 days	Homocysteine levels at rest and after acute exhaustive exercise	**Body Composition:** Not measured. **Performance:** Not measured. **Recovery/Fatigue:** Cr did *not* reduce the rise in plasma homocysteine caused by acute exhaustive exercise—both Cr and placebo groups showed similar increases in homocysteine post-exercise.
**Williams et al., 2014**	16 male amateur soccer players	DB, PC, matched-pairs; randomisation not reported (ROBINS-I)	Cr monohydrate 20 g/day for 7 days	ball-sport endurance and sprint test, Yo-Yo IR, 12 m and 20 m sprint, circuit time, vertical jump, body mass, HR and RPE	**Body Composition:** No significant between-group difference was observed in body mass. Performance: No detectable between-group benefit of creatine supplementation was reported for the assessed sprint, endurance, circuit, or vertical-jump outcomes. Recovery/Fatigue: No statistically significant between-group differences in HR were observed.
**Claudino et al., 2014**	14 male elite soccer players	R, DB, PC	20 g/day of Cr monohydrate for 1 week (4 × 5 g/day), followed by 5 g/day for the next 6 weeks	lower-limb muscle power (CMJ performance), body mass	**Body Composition:** No significant changes in body mass. **Performance:** Jumping performance slightly improved in the Cr group and declined in the placebo group, though the difference was not statistically significant. However, significantly more participants in the placebo group showed reduced performance after training. **Recovery/Fatigue:** Not measured.
**Shojaeian et al., 2014**	20 male soccer players	R, DB, PC	Cr monohydrate 20 g/day for 7 days	sprint test, preplanned change-of-direction tests with and without the ball and dribbling, and Yo-Yo IR	**Body Composition:** Not measured. Performance: Favourable findings were reported for sprint, dribbling, and preplanned change-of-direction performance with and without the ball; however, the extracted summary did not clearly establish whether all outcomes represented significant between-group treatment effects. **Recovery/Fatigue:** HR and systolic and diastolic blood pressure decreased after one week of creatine use; the comparative statistical basis was not clearly described in the extracted summary.
**Deminice et al., 2016**	13 young soccer players	R, DB, PC	Cr monohydrate 0.3 g/kg/day for 7 days	TBW (via a deuterium oxide dilution method and BIA formulas)	**Body Composition:** TBW and body mass increased within the creatine group, as assessed using deuterium oxide dilution and several BIA equations; however, the comparative treatment effects were not clearly reported in the extracted summary. **Performance:** Not measured. **Recovery/Fatigue:** Not measured.
**Ramírez-Campillo et al., 2016**	20 young female soccer players	R, DB, PC	Cr monohydrate: 20 g/day for 7 days, then 5 g/day for 5 weeks	Body mass, squat jump, CMJ, 20-m sprint test and RAST, 20-cm and 40-cm drop jump reactive strength indexes, peak jump power and peak jump power load test, unilateral 20-cm drop jump reactive strength indexes (right and left leg), change-of-direction speed test (i.e. Illinois test) and 20-m multistage shuttle run tests.	**Body Composition:** Body mass and BMI significantly increased in the Cr group. **Performance:** Peak jump power load, squat jump height, 40 cm reactive strength index, and RAST mean sprint time improved in the Cr group compared to placebo, while no significant differences were observed in other tests. **Recovery/Fatigue:** Not measured.
**Ateş et al., 2017a**	13 young male soccer players	NR, repeated-measures	Cr monohydrate 0.3 g/kg/day for 5 days	RST (e.g. 6 × 35 m sprint time, FI)	**Body Composition:** Not measured. Performance: A favourable finding was reported for one repeated-sprint measure, whereas total repeated-sprint time did not improve; the comparative statistical basis was not clearly described in the extracted summary. **Recovery/Fatigue:** FI was lower after creatine supplementation; however, the comparative treatment effect was not clearly reported.
**Ates et al., 2017b**	20 male soccer players	NR, repeated-measures	Cr monohydrate 0.3 g/kg/day for 5 days	Body composition; Illinois change-of-direction test	**Body Composition:** The creatine group gained more body mass than the comparator group, with no significant change in body fat. **Performance:** Illinois test performance improved more in the creatine group; this outcome represents preplanned change-of-direction performance rather than reactive agility. **Recovery/Fatigue:** Not measured.
**Yáñez-Silva et al., 2017**	19 male elite soccer players	R, DB, PC	Cr monohydrate 0.3 g/kg/day for 14 days	30-s Wingate anaerobic test, PP, MP, FI, and total work	**Body Composition:** Not measured. **Performance:** PP and MP increased within the Cr group, but group × time interactions were not significant, providing no detectable comparative benefit for these outcomes. Total work was higher in the Cr group post-supplementation, although this isolated postintervention difference is difficult to interpret without a significant interaction. **Recovery/Fatigue:** No significant change in FI was reported after creatine or placebo supplementation.
**Rodríguez-Ramírez et al., 2018**	15 male college soccer players	R, DB; creatine-timing comparison (both groups received creatine)	All participants: Cr 20 g/day for 5 days, then 5 g/day for 3 weeks; randomised comparison of Cr before vs after training	Isometric strength in biceps, back, legs and forearms	Body Composition: Not measured. Performance: Both groups received creatine; the randomised comparison tested timing (before vs after training), not creatine efficacy versus no creatine. No significant between-group strength differences were reported at the end of the intervention. Recovery/Fatigue: Not measured.
**Radzimiński et al., 2019**	15 adult soccer players	Active-comparator controlled study; allocation method not verifiable as randomised (ROBINS-I)	Cr monohydrate vs Cr malate, 10 g/day on training days (5 g fasted, 5 g post-exercise); 5 g/day on rest days	aerobic (PWC 170 test) and anaerobic (Wingate anaerobic test) capacity	**Body Composition:** Not measured. **Performance:** VO₂max, PWC170, and total anaerobic work improved within the creatine-monohydrate group but not the creatine-malate group. Because both groups received a creatine formulation and no clear between-group comparison was reported, this study informs formulation comparison rather than creatine-versus-placebo efficacy. **Recovery/Fatigue:** Not measured.
**Simpson et al., 2019**	19 elite soccer players	R, DB, PC	Cr monohydrate 0.3 g/kg/day for 1 week, then 5 g/day for 7 weeks	Airway inflammation (assessed by exhaled nitric oxide, F_E_NO) and airway responsiveness (to dry air hyperpnoea), Body composition (skinfold measurement)	**Body Composition:** No between-group differences were observed in body mass and skinfold thickness between the Cr and placebo groups. **Performance:** Not measured. **Recovery/Fatigue:** No statistically significant changes were found in FENO or FEV₁ response to dry air hyperpnoea.
**Azevedo et al., 2019**	8 male elite soccer players	NR crossover, SB, PC; fixed placebo→creatine sequence (ROBINS-I)	Cr monohydrate 0.3 g/kg/day for 7 days	HIIT tests, VGRF and EMG, HR, RPE and lactate concentration	**Body Composition:** Not measured. **Performance:** Creatine-period testing showed lower impact-related VGRF and altered EMG responses during HIIT; however, placebo was always administered before creatine, so treatment and period effects cannot be separated cleanly. **Recovery/Fatigue:** No clear comparative benefit for HR, RPE, or lactate was established. Fixed sequence and possible residual/period effects were considered in ROBINS-I.
**Wand et al., 2019**	14 analysed soccer players (7/group; abstract reports 20)	R, DB, PC; source-level sample-size inconsistency	Cr 20 g/day for 4 weeks	Wingate anaerobic test, blood lactate	**Body Composition:** Not measured. **Performance:** The Methods and tables analyse 14 participants (7/group), despite the abstract reporting 20. Peak anaerobic power was reported to improve after creatine, but the comparative effect was not consistently documented. **Recovery/Fatigue:** Blood lactate was reported lower 3 minutes post-exercise after creatine; interpretation is limited by the source-report inconsistency.
**Zajac et al., 2020**	20 elite soccer players	R, PC	Mg- Cr chelate 0.07 g/kg/day for 16 weeks	RAST, first and sixth 35 m sprint length, AP, MP, lactate, acid–base equilibrium pH, bicarbonates	**Body Composition:** Not measured. Performance: Total time, absolute power, mean power, and first- and sixth-sprint performance improved within the creatine condition, whereas no significant changes were reported in placebo; however, the extracted summary did not clearly establish a significant between-group treatment effect. **Recovery/Fatigue:** Post-exercise lactate, resting pH, and bicarbonate increased, whereas post-exercise pH decreased after creatine supplementation; the comparative statistical basis was not clearly described in the extracted summary.
**Mor et al., 2022**	24 male amateur soccer players	R, SB, PC	Cr 2 g/day for 7 days	RAST (six consecutive 35-metre sprints by giving 10-second breaks), Ball speed measurement, blood lactate, HR	**Body Composition:** Not measured. **Performance:** Anaerobic capacity: Mean power output during the RAST was reported to increase after creatine supplementation; however, the extracted summary did not fully describe the comparative analysis. After fatiguing exercise, the creatine group maintained kicking speed better than placebo. **Recovery/Fatigue:** No significant effect was reported for post-exercise recovery HR or blood lactate. FI did not change within the creatine group; a comparative treatment effect was not clearly reported.
**Pedrosa et al., 2022**	16 amateur soccer players	R, PC	Cr: 20 g/day for 7 days, then 5 g/day for 3 weeks	Anthropometric and hydration assessment (BIA), BRVJ, RAST, Wingate anaerobic test	**Body Composition:** Intragroup analysis showed significant increases in body mass, BMI, and limb circumferences in the Cr group; however, no significant between-group differences were observed in anthropometric measures, body composition, or hydration status. **Performance:** Cr supplementation led to significant within-group improvements in maximum power, relative power, and average power in both the RAST and BRVJ tests, but no significant between-group differences were observed. However, in the Wingate anaerobic test, significant between-group differences were found, with the Cr group showing greater improvements in power-related outcomes. **Recovery/Fatigue:** FI in the RAST and BRVJ tests, as well as tiredness level (%) in the Wingate anaerobic test, showed no significant changes following Cr or placebo supplementation.
**Valenta et al., 2022**	16 adolescent soccer players	R, DB, PC	Cr monohydrate 20 g/day for 5 days	Vertical jump tests (vertical squat jumps and CMJ), Single running sprint test (three trials of the 10-m sprint test, with 90-s rest), single cycling sprint test (two single 6-s cycling sprints on a bicycle ergometer), body composition (BIA)	**Body Composition:** Body mass and BMI increased within the creatine group. No significant between-group differences were reported for lean mass, body fat, TBW, intracellular water, or extracellular water. **Performance:** Cr supplementation had no significant effect on performance outcomes, including peak and mean power during cycling sprints, squat jump, 10-m sprint, or vertical jump tests. **Recovery/Fatigue:** Cr did not alter post-exercise blood lactate levels or FI.
**Masodsai et al., 2022**	17 male soccer players	R, DB, crossover, PC	Cr monophosphate: single 5 g dose in a glucose-electrolyte sports drink	Cardiac, respiratory, and metabolic functions, lactate, glucose	**Body Composition:** Not measured. **Performance:** All participants completed the beverage conditions in randomised order; a single 5-g creatine dose produced no detectable sprint-performance benefit compared with control. **Recovery/Fatigue:** No significant differences were observed in lactate or cardiorespiratory/metabolic recovery outcomes. Crossover-specific RoB 2 was used.
**Jauregui et al., 2023**	16 male youth soccer players	R, DB, PC; same parent trial as Jauregui 2024	Cr monohydrate: 0.3 g/kg/day for 7 days, then 0.03 g/kg/day for 21 days	Body mass RAST (six 35-m sprints, 10 s rest)	**Body Composition:** Body mass increased within the creatine group over 4 weeks but not placebo; a significant between-group treatment effect was not clearly reported. **Performance:** No significant improvement in 35-m sprint times was observed in either group from pre to post. Both groups exhibited fatigue across the six sprints (6th sprint slower than 1 st in both). **Recovery/Fatigue:** Not measured.
**Yapıcı et al., 2023**	21 healthy male soccer players	R, DB, crossover/repeated-condition; washout not reported	Cr 20 g for 5 days, then 5 g/day for 10 days, or 0.3 g/kg/day for 15 days	Vertical jump, 10-30 m sprint test and change-of-direction test (Zig-Zag test)	**Body Composition:** Not measured. **Performance:** Participants completed multiple creatine/placebo conditions using a repeated-condition design. The higher-dose loading protocol showed within-condition improvements, but sequence and washout reporting were insufficient to exclude period/carryover effects. **Recovery/Fatigue:** Not measured. Crossover-specific RoB 2 was used.
**Jauregui et al., 2024**	16 male youth soccer players	R, DB, PC; same parent trial as Jauregui 2023	Cr monohydrate: 0.3 g/kg/day for 7 days, then 0.03 g/kg/day for 21 days	RAST-derived power and sprint velocity (mean and peak)	**Body Composition:** Body mass increased within the creatine group over 4 weeks but not placebo; a significant between-group treatment effect was not clearly reported. **Performance:** Cr had no effect on peak power or peak sprint velocity in the repeated-sprint test compared to placebo. There was a trend toward higher mean power output and mean sprint velocity in Cr group, though not statistically significant. **Recovery/Fatigue:** Not measured.
**Ojeda et al., 2024a**	28 young soccer players	R, DB, factorial/parallel; same parent cohort as Ojeda 2024b	Cr monohydrate 0.3 g/kg/day for 14 days; Caffeine 0.3 mg/kg/day	RSA test; strength (1RM in HBS)	**Body Composition:** Not measured. **Performance:** After 2 weeks, no significant differences in repeated 30-m sprint performance (RSA test) or squat outputs were found among the four groups (Cr + caffeine, Cr alone, caffeine alone, placebo). **Recovery/Fatigue:** Cr did not significantly alter fatigue in this short period when combined with a pre-activation regimen.
**Ojeda et al., 2024b**	28 young soccer players	R, DB, PC; same parent cohort as Ojeda 2024a	Cr monohydrate 0.3 g/kg/day for 14 days	RSA test; strength (1RM in HBS), muscle power development after fatigue induction, velocity and power generated in HBS, force, rate of force development in HBS, FI, and total time in the RSA test	**Body Composition:** Not measured. **Performance:** No significant group differences were observed in bar velocity, power, force, or RFD during the HBS following Cr supplementation compared to placebo. **Recovery/Fatigue:** No significant differences were found between groups in FI and total time during the RSA test.

Study/report counting note: This table contains 45 eligible reports representing 41 unique studies. Four report sets were consolidated at the study level because they arose from the same or strongly overlapping parent cohorts: Deminice et al. [32,33], Jauregui et al. [39,40], Ojeda et al. [45,46], and Ranjbar/Tabesh [52,54]. Both reports in each set are retained for outcome-level traceability, but participants and risk-of-bias assessments are counted once. The two Faramarzi et al. [36] entries involved different participant samples and supplementation protocols and were retained as separate studies. Chen et al. [29] was excluded after source-level verification because the available report did not describe an eligible comparator. Studies with reported non-random allocation or for which a random allocation procedure could not be verified from the available source report were assessed conservatively using ROBINS-I; this included Smart et al. [14], for which matching/assignment was described without a verifiable random allocation procedure.

Abbreviations: R = randomised; NR = non-randomised; DB = double-blind; SB = single-blind; PC = placebo-controlled; CrO = crossover; ND = not determined; Cr = creatine; CHO = carbohydrate; 1RM = one-repetition maximum; FM = fat mass; FFM = fat-free mass; BFP = body fat percentage; CK = creatine kinase; CK-MB = creatine kinase myocardial isoform; IL-6 = interleukin-6; BIA = bioelectrical impedance analysis; RAST = Running-based Anaerobic Sprint Test; CMJ = countermovement jump; FI = fatigue index; SBJ = standing broad jump; VO₂max = maximal oxygen uptake; Yo-Yo IR = Yo-Yo intermittent recovery test; HBS = half-back squat; FEV₁ = forced expiratory volume in 1 second; HIIT = high-intensity interval training; VGRF = vertical ground reaction force; EMG = electromyography; HR = heart rate; RPE = rating of perceived exertion; VM = vastus medialis; VL = vastus lateralis; GL = gastrocnemius lateralis; TTE = time to exhaustion; BUN = blood urea nitrogen; AST = aspartate aminotransferase; ALT = alanine aminotransferase; γGT = gamma-glutamyl transferase; TBW = total body water; CSA = cross-sectional area; CMJT = countermovement jump test; IET = intermittent endurance test; RST = repeated sprint test; RSA = repeated sprint ability; BRVJ = Bosco repeated vertical jump.

Interpretive note: timing-only and formulation-only comparisons are retained for completeness but are not interpreted as creatine-versus-no-creatine efficacy comparisons. Participant totals elsewhere in the review use the analysed sample when this was the clearest denominator supporting the reported outcomes (e.g. Wand n=14); source-report discrepancies are explicitly noted.

The two Faramarzi et al. reports published in 2009 involved different samples and supplementation protocols and were therefore treated as separate studies. Four other report sets were consolidated because they arose from the same or strongly overlapping parent cohorts: Deminice et al. [32,33], Jauregui et al. [39,40], Huerta Ojeda et al. [45,46], and Ranjbar/Tabesh [52,54]. Both reports from each set were retained in Table 1 for outcome-level traceability, but participants and methodological risk-of-bias summaries were counted once. Chen et al. [29], which had initially been extracted as eligible, was excluded after source-level verification because the available report described a single creatine-treated cohort without an eligible placebo, control, or comparison condition. The final review therefore included 45 reports representing 41 unique studies.

The duration of creatine supplementation protocols varied across studies, with acute protocols lasting 4–7 days (typically using a loading dose of 20 g/day or 0.3 g/kg/day) and chronic protocols ranging from 14 days to 28 weeks with daily doses of 3–10 g. However, one study assessed the effects of a single creatine dose [62]. The majority of interventions used creatine monohydrate, with a few studies including creatine malate [48] or Mg-creatine chelate [60].

At the unique-study level, 28 studies used randomised parallel-group designs, 2 used randomised crossover/repeated-condition designs, and 11 were non-randomised or had allocation that could not be verified as randomised. Standard RoB 2 domains were used for the randomised parallel studies, crossover-specific RoB 2 domains for the randomised crossover/repeated-condition studies, and ROBINS-I domains for the remaining controlled studies. Across the evidence base, outcomes included sprint and repeated-sprint performance, jumping, Wingate-derived anaerobic power, strength, fatigue indices, biochemical markers, and anthropometric or body-composition measures.

Among the 28 randomised parallel-group studies assessed with standard RoB 2, 12 were judged at low overall risk of bias, 2 had some concerns, and 14 were judged at high overall risk. The most frequent concerns involved missing outcome data and selection of the reported result, whereas all 28 studies were judged at low risk for outcome measurement (Figure 2A–B). The two randomised crossover/repeated-condition studies were assessed separately with crossover-specific RoB 2: Masodsai et al. was judged as having some concerns, whereas Yapıcı et al. was judged at high overall risk, principally because period/carryover effects could not be excluded and sequence/washout reporting was incomplete (Figure 2C). All 11 studies assessed with ROBINS-I were judged at serious overall risk, with confounding representing the most consistent concern and additional moderate, serious, or no-information judgements in other domains (Figure 3). Azevedo et al. was assessed with ROBINS-I because the available report used a fixed placebo-to-creatine sequence rather than a randomised treatment sequence [21]. Because period and treatment were not randomised, this study was not treated as a randomised crossover trial; possible period and residual-treatment effects were considered within the non-randomised risk-of-bias assessment.

Table 2 provides a structured overview of the three outcome domains. In brief, body mass often increased, particularly after loading protocols, whereas findings for lean mass, fat mass, hydration, performance, and recovery/fatigue were less consistent.

**Table 2.** Descriptive narrative synthesis by outcome domain.

Outcome Domain	Studies (*n*)	Participants (*n*)	Summary of Findings
Body Composition	23	460	Body-mass increases were frequently reported following creatine loading; however, not all studies demonstrated a significant comparative treatment effect. Findings for fat-free mass and fat mass were inconsistent. Counts are based on unique cohorts; overlapping reports were counted once.
Performance	36	659	Some eligible studies reported favourable comparative findings for repeated-sprint ability, anaerobic power, and muscular strength, but findings were heterogeneous and several studies reported null, within-group-only, or statistically unclear effects. Timing/formulation-only comparisons were not interpreted as creatine-versus-control efficacy evidence.
Recovery/Fatigue	26	544	Comparative evidence for recovery or fatigue benefits was limited and inconsistent. Overlapping Deminice, Ranjbar/Tabesh, and Ojeda reports were counted once at the cohort level; participant totals also use the analysed *n* = 14 reported in the Wand Methods/tables.

Counting note: Study and participant totals are based on unique cohorts within each domain. Multiple reports from the same parent cohort were counted once; this applies to Deminice [32,33], Jauregui [39,40], Ojeda [45,46], and Ranjbar/Tabesh [52,54] where relevant. Chen [29] was excluded because no eligible comparator was described. For Wand, *n* = 14 was used because the Methods and outcome tables analysed 7 participants per group, despite the abstract reporting *n* = 20.

Synthesis rule: findings were categorised as favourable comparative evidence only when a between-group difference or group-by-time interaction supported creatine; explicit nonsignificant comparative tests were categorised as null; within-group-only changes or reports without an interpretable comparative statistic were categorised as unclear/within-group only. Timing/formulation-only comparisons were not treated as creatine-versus-control efficacy evidence. No inferential vote-counting test or numerical weighting by study size was used; high-risk and non-randomised evidence was interpreted cautiously.

### 3.2. Effects of creatine supplementation on performance

The included studies used a wide range of performance tests. For clarity, we grouped these outcomes into sprint and repeated-sprint performance, anaerobic power, jump performance, change-of-direction ability, muscular strength, technical skills, and intermittent high-intensity endurance.

#### 3.2.1. Effects of creatine supplementation on sprint and repeated-sprint performance

Sprint and repeated-sprint outcomes were evaluated across distances of 10–100 m. Findings were heterogeneous: some studies reported favourable comparative effects, whereas others reported within-group improvements without significant between-group effects or no detectable benefit. For example, Mujika et al. reported within-group improvements in repeated-sprint performance, with total time over 6 × 15-m sprints decreasing in the creatine group but not in the placebo group. However, the available report did not clearly establish a significant between-group treatment effect [15]. Cox et al. reported favourable differences in selected sprint and preplanned change-of-direction blocks, but average performance across the protocol did not differ significantly between conditions [31]. Ostojic et al. reported significantly better post-supplementation sprint-power performance in the creatine group than in the placebo group [16]. In a longer trial, Imamoğlu et al. reported faster repeated-sprint times in the creatine group after 20 g/day for 1 week followed by 5 g/day for 3 weeks; however, the allocation method was not determined [38]. Faramarzi et al. reported an improvement in 60-m sprint time after 10 g/day for 10 days, although the extracted summary did not clearly establish a significant between-group effect; in a separate Faramarzi et al. study, 3 g/day for 6 days was associated with improved Running-based Anaerobic Sprint Test (RAST) power outcomes compared with control [36,61]. Other studies reported favourable findings for selected sprint outcomes [25,32,43,49,50,59,60], although the statistical basis and consistency of these effects varied across studies. Tartibian et al. reported within-group improvements in sprint times and knee-extension strength, but no significant between-group differences were detected; therefore, this trial provided no detectable comparative evidence of benefit [53].

Several studies reported no significant between-group benefit or provided only within-group or statistically unclear findings. For example, Jauregui et al. reported a tendency toward higher mean power output and mean sprint velocity in the RAST test following creatine supplementation, though the differences were not statistically significant [40]. Pedrosa et al. found no significant between-group differences in RAST maximum, relative, or average power. Although these variables improved within the creatine group, the within-group changes do not establish a supplementation effect [47]. In another study, Smart et al. showed that 24 g/day of creatine for 6 days did not enhance performance in 30 maximal 20-m sprints [14]. Likewise, Esmaeeli et al. found no improvement in sprint performance following a 5-day creatine loading protocol compared to placebo [35]. Several other studies have also reported no ergogenic effect of creatine supplementation on sprint performance [17,35,40,45,56,62].

#### 3.2.2. Anaerobic power

Anaerobic power was most frequently evaluated using the Wingate anaerobic test. Findings for anaerobic-power outcomes were mixed. Some trials reported significant comparative benefits for selected Wingate outcomes, whereas others reported within-group changes without significant interactions or did not provide a valid between-group comparison. For example, Ünal et al. reported within-group increases in mean power, minimum power, and total work after creatine supplementation. Because the reported summary does not establish a significant between-group treatment contrast, the comparative effect remains uncertain [55]. In a longer trial, Wand et al. reported an increase in peak anaerobic power following creatine supplementation at 20 g/day for 4 weeks; however, a significant between-group treatment effect was not clearly reported [57]. Pedrosa et al. also reported significant between-group differences in power-related variables after creatine supplementation (20 g/day for 7 days, followed by 5 g/day for 3 weeks) [47]. Yáñez-Silva et al. reported increases in peak and mean power within the creatine group; however, the group × time interactions were not significant, providing no detectable comparative benefit for these outcomes. Total work was higher in the creatine group at postintervention, although the interpretation of this isolated postintervention difference is uncertain [58]. Radzimiński et al. reported within-group improvements in the creatine monohydrate group but not in the creatine malate group; however, because no between-group comparison was reported, the comparative effect of the two creatine formulations remains unclear [48].

#### 3.2.3. Jump performance

Findings regarding jump performance were mixed. Vertical jump ability was assessed most commonly using the squat jump (SJ) and countermovement jump (CMJ), while one study used the triple-broad jump. Some studies reported favourable comparative findings, whereas others reported only within-group changes or no detectable between-group benefit. Mujika et al. also reported a within-group improvement in vertical-jump performance after a 6-day loading protocol, but the comparative effect relative to placebo was not clearly established [15]. Ostojic et al. reported a significant favourable post-supplementation difference in vertical-jump height between creatine and placebo [16]. In a 3-week study, Imamoğlu et al. reported greater improvement in vertical-jump performance in the creatine group than in the comparator group; however, the allocation method was not determined [38].

Conversely, Ünal et al. found no changes in vertical jump following 0.25 g/kg/day of creatine monohydrate for 4 days. Esmaeeli et al. also reported no significant change in CMJ height in any group after 5 days of creatine loading [35]. Comparable null results were observed in other studies [36,56]. Claudino et al. and Pedrosa et al. reported no significant between-group benefit of creatine supplementation on jump performance. Within-group numerical or statistically significant improvements were observed in the creatine conditions, but these findings do not demonstrate a treatment effect [30,47].

#### 3.2.4. Change-of-direction ability

Only a small number of studies assessed change-of-direction (COD) performance, and their findings were generally null or inconsistent. Ateş et al. reported a small, nonsignificant change in Illinois COD test performance after creatine supplementation; Ramírez-Campillo et al. found no significant between-group difference after 5 weeks; and Yapıcı et al. found no improvement in Zig-Zag COD performance after 15 days of supplementation. These tests involved preplanned directional changes and should therefore be interpreted as measures of COD ability rather than reactive agility.

#### 3.2.5. Muscular strength outcomes

Findings for muscular-strength outcomes were mixed, and interpretation depended on whether reports provided valid between-group comparisons rather than within-group changes alone. Larson-Meyer et al. found that 12 weeks of creatine supplementation led to greater improvements in bench press (weeks 0–5) and full squat strength (weeks 5–13) compared to placebo [41]. Similarly, İmamoğlu et al. reported greater improvements in leg strength after 4 weeks of creatine intake compared to placebo [38]. Faramarzi et al. reported an improvement in leg strength following 10 days of creatine supplementation; however, the extracted summary did not clearly establish a significant between-group treatment effect [36], while Rodríguez-Ramírez et al. compared creatine timing (before versus after training) rather than creatine versus no creatine; no clear between-group timing advantage was demonstrated [63]. However, some other studies failed to show improvements [45,46,53].

#### 3.2.6. Technical skill tests

Findings for soccer-specific technical outcomes were mixed. A few studies assessed kicking and dribbling performance. Cox et al. measured precision ball-kicking accuracy following a 6-day creatine loading protocol and found no significant changes [31]. Mor et al. assessed ball speed after creatine supplementation (2 g/day for 7 days) and reported that the creatine group maintained kicking speed better than the placebo group [44]. Mohebbi et al. reported no significant change in shooting accuracy following creatine intake (20 g/day for 7 days) [43].

Regarding dribbling performance, Ostojic et al. reported a significant favourable post-supplementation difference in dribble-test time between creatine and placebo [16]. Shojaeian et al. also reported favourable dribbling findings after 20 g/day for 7 days, but the comparative statistical basis was not clearly described [50]. Similarly, Mohebbi et al. reported an improvement in dribbling performance within the creatine group; however, the comparative treatment effect was not clearly documented [43].

#### 3.2.7. Intermittent high-intensity endurance and performance

Given the intermittent nature of soccer, tests such as the Yo-Yo Intermittent Recovery Test and the Intermittent Sprint Endurance Test were commonly used to assess endurance capacity. Endurance performance is considered one of the key conditioning parameters in soccer players. Across the studies assessing endurance, creatine supplementation did not improve endurance performance. For instance, Biwer et al. found no improvement in intermittent endurance performance on a treadmill following creatine monohydrate supplementation at 0.3 g/kg/day for 6 days [27]. Similarly, Ostojic et al. reported no within- or between-group changes in endurance performance after a 1-week creatine loading protocol [16]. Machado et al. also observed no significant improvement in treadmill graded exercise test (GXT) duration following creatine monohydrate supplementation at 0.6 g/kg/day for 5 days compared to placebo [42].

### 3.3. Effects of creatine supplementation on recovery and fatigue

Recovery- and fatigue-related outcomes were reported in 26 of the 41 unique studies, represented by 29 reports. Measures included fatigue index (FI), blood lactate, inflammatory markers, muscle-damage markers such as creatine kinase (CK) and lactate dehydrogenase (LDH), heart rate (HR), and ratings of perceived exertion (RPE).

#### 3.3.1. Fatigue index and repeated sprint fatigue

Several studies assessed fatigue indices using the RAST, Wingate test, or other anaerobic performance measures. Evidence for fatigue-index outcomes was inconsistent, and few studies reported clear comparative benefits. Ünal et al. reported a within-group reduction in fatigue index during the Wingate test; however, a significant between-group treatment effect was not clearly established [55]. Ateş et al. also reported a favourable fatigue-index finding, but the comparative statistical basis was not clearly described [25]. Mujika et al. reported less fatigue across successive sprints in the creatine group; however, a significant between-group treatment effect was not clearly demonstrated [15]. Overall, fatigue-index findings were inconclusive, with several trials reporting no significant differences between the creatine and placebo groups. Esmaeeli et al. reported no significant change in FI in either group [35], and similarly, Deminice et al. found no between-group differences [32]. Yáñez-Silva et al. also observed no significant changes in FI after either creatine or placebo supplementation [58]. These null findings were consistent with results from several other included studies [44–47].

#### 3.3.2. Post-exercise blood lactate, acid–base balance and fatigue-related metabolites

Ten studies evaluated post-exercise blood lactate concentrations. Findings regarding blood lactate responses were variable across the included studies, with several trials reporting no significant effects of creatine supplementation. For example, Smart et al. found no significant differences in plasma lactate concentrations after 24 g/day of creatine for 6 days [14], while Mujika et al. reported no significant changes in after the repeated sprint test (RST) and intermittent endurance test (IET) lactate levels [15]. Similar null effects were observed in several other studies [21,27,32,44,56,62]. In contrast, Cox et al. reported that creatine supplementation significantly reduced post-exercise blood lactate in certain blocks and slightly increased blood pH in the early blocks [31]. Wand et al. reported lower blood lactate concentrations 3 minutes after exercise following supplementation; however, a significant between-group treatment effect was not clearly reported [57]. Conversely, Ünal et al. found an increase in post-exercise lactate compared to baseline in the creatine group [55]. Likewise, Zajac et al. reported within-condition changes in post-exercise lactate, resting pH, bicarbonate, and post-exercise pH; however, the comparative statistical basis was not clearly described [60].

A few studies assessed exercise- and fatigue-related metabolites, including uric acid [14,28,42], urea [42], ammonia [15,42], blood urea nitrogen (BUN) [28], glucose [28,62], liver markers (total proteins and albumin, aspartate aminotransferase (AST), alanine aminotransferase (ALT), gamma-glutamyl transferase (γGT) [28], urinary creatinine [28], and hypoxanthine [14]. Most assessed metabolites showed no clear between-group differences. Reductions in glucose and urea were reported in individual studies, although the comparative statistical basis of these findings was not consistently established [28,42].

#### 3.3.3. Markers of muscle damage, inflammation and oxidative stress

Five studies assessed creatine kinase (CK), LDH, or inflammatory markers. Cancela et al. reported increased CK levels after 7 weeks of creatine supplementation [28]. Similarly, Atashak and Jafari found significantly greater increases in CK and creatine kinase-MB (CK-MB) post-exercise in the creatine group compared to placebo following 1 week of creatine loading [24]. In contrast, Faramarzi et al. observed no change in CK levels after 6 days of creatine loading [36]. Deminice et al. reported that creatine supplementation inhibited exercise-induced increases in LDH activity following a 1-week loading phase [32]. However, two other studies found no significant changes in LDH activity [24,36]. Regarding the inflammatory response, Deminice et al. reported that creatine supplementation inhibited exercise-induced increases in inflammatory cytokines (tumour necrosis factor alpha (TNF-*α*) and C-reactive protein (CRP)) following a 1-week loading phase [32]. In contrast, Simpson et al. found no statistically significant changes in airway inflammation, as assessed by exhaled nitric oxide (fractional exhaled nitric oxide (FENO)) [51].

Two reports from the same University of Mazandaran parent cohort described reductions in plasma homocysteine after creatine supplementation, although the comparative statistical basis was not consistently clear. Tabesh et al. reported a reduction in post-exhaustive exercise homocysteine after 5 days of creatine loading [52]. Ranjbar et al., which appears to represent the creatine-versus-control subset of the same parent cohort, reported reductions in plasma homocysteine at rest and after exhaustive exercise; however, the comparative statistical basis was not clearly described [54]. However, Deminice et al. observed that a 7-day creatine loading protocol did not attenuate the rise in plasma homocysteine induced by acute exhaustive exercise; both creatine and placebo groups showed similar post-exercise increases [33]. Regarding oxidative stress markers, only one study by Deminice et al. assessed this outcome and found that creatine had no effect, with antioxidant enzyme activity increasing similarly in both the creatine and placebo groups [32].

#### 3.3.4. Heart rate and ratings of perceived exertion

Heart rate recovery was assessed in a limited number of studies, with most reporting no significant differences following creatine supplementation. Four out of six studies found that creatine did not alter HR [17,21,44,62]; for example, Williams et al. observed no statistically significant changes after a 1-week loading phase [17], while Azevedo et al. and Mor et al. similarly reported no significant effects on post-exercise recovery HR after 7 days of supplementation [21,44]. Conversely, Cox et al. found that creatine significantly reduced post-exercise HR in certain testing blocks [31], and Shojaeian et al. reported reductions in HR and systolic and diastolic blood pressure after one week of creatine use; however, the comparative statistical basis was not clearly described [50]. Regarding perceived exertion (RPE), all five studies that evaluated this parameter found no significant changes following creatine supplementation [17,21,27,31,62].

### 3.4. Effects of creatine supplementation on body composition

Of the 41 unique studies, 23 (represented by 24 reports) assessed at least one body-composition outcome. The most commonly reported measures were body mass, lean mass, fat mass, body mass index (BMI), and total body water (TBW).

#### 3.4.1. Body mass, BMI, and total body water

Body mass increases were frequently reported after creatine supplementation, although the strength of comparative evidence varied across studies. At the unique-study level, 16 of 23 studies [14,15,20,26–28,31,32,34,35,37–40,43,54,56] reported an increase in body mass within the creatine condition. However, not all studies demonstrated a statistically significant difference in body-mass change between creatine and comparator groups, and these counts should therefore be interpreted descriptively rather than as 17 confirmed treatment effects. At the report level, 17 reports described a body-mass increase. Among these reports, the reported weight gain ranged from 0.4 kg [15,28] to 3.6 kg [38]. Of the 17 reports, 8 reported weight gains of ≥ 1 kg [14,27,34,37,38,40,43,54], 5 reported gains of < 1 kg [15,26,28,31,56], and 4 did not report the exact amount of weight change [20,35,39,49]. Of the four studies that reported BMI, three observed an increase [49,54,56], while one reported no change [47].

Only three studies evaluated TBW directly. Deminice et al. [34] reported increases in TBW measured by deuterium oxide dilution and selected bioelectrical impedance analysis (BIA) equations; however, the comparative treatment effect was not clearly reported in the extracted summary [34]. In contrast, Valenta et al. found no significant between-group changes in TBW or its compartments (intracellular and extracellular water) despite weight gain in the creatine group. Similarly, Pedrosa et al. reported no significant between-group differences in hydration status [47].

#### 3.4.2. Fat mass and lean mass

Some studies reported increases in lean mass or muscle mass, particularly in longer-duration trials; however, the strength of the comparative evidence varied. In a 28-week conference report, Gallo et al. described increases in body mass, muscle mass, and calf cross-sectional area; because the full methodological report could not be retrieved, random allocation and the comparative statistical basis could not be verified, and this study was therefore interpreted cautiously [20]. Larson-Meyer et al. observed that both groups increased fat-free mass and body mass with training, but 13 weeks of creatine supplementation did not produce additional gains beyond those achieved with training alone [41].

In shorter studies, Mohebbi et al. reported increases in body mass and lean body mass within the creatine group after 7 days, but a significant between-group effect was not clearly documented. Valenta et al. found no significant between-group difference in lean mass after 5 days of supplementation, and Faramarzi et al. likewise reported no significant change in fat-free mass after 10 days.

Results regarding body fat were mixed. Among nine studies assessing body fat, three reported reductions in fat mass after creatine use; however, the comparative statistical basis varied across studies [20,38,54], whereas others reported no significant changes [26,32,36,41,43,56].

### 3.5. Certainty of evidence

GRADE certainty was rated as very low for all three broad outcome domains. Body composition was downgraded because of methodological limitations, suspected publication bias, inconsistency, and imprecision. Performance was also rated very low because of methodological limitations, suspected publication bias, inconsistency, and imprecision. Recovery/fatigue evidence was rated very low because of methodological limitations, suspected publication bias, substantial inconsistency, and imprecision (Table 3).

**Table 3.** Domain-level GRADE certainty assessment for body composition, performance, and recovery/fatigue outcomes.

Outcomes	Methodological limitations of the studies	Inconsistency	Imprecision	Indirectness	Publication Bias	Quality of evidence
Body Composition	Serious	Serious	Serious	Not serious	Suspected	Very Low
Performance	Serious	Serious	Serious	Not serious	Suspected	Very Low
Recovery/Fatigue	Serious	Very serious	Serious	Not serious	Suspected	Very Low

These are domain-level summary certainty judgements. Certainty was anchored to randomised evidence; non-randomised studies were treated as supportive/contextual evidence and did not increase certainty. Methodological limitations were informed by the study-level RoB-domain summaries (28 randomised parallel, 2 randomised crossover/repeated-condition, and 11 non-randomised/allocation-unverifiable studies). Because the domains contain heterogeneous outcomes and no pooled estimate was available, the ratings should not be interpreted as outcome-specific GRADE certainty estimates.

Downgrade rationale: Body composition - serious methodological limitations, serious inconsistency, serious imprecision, no serious indirectness, and suspected publication bias. Performance - serious methodological limitations, serious inconsistency, serious imprecision (small heterogeneous trials without a precise pooled estimate), no serious indirectness, and suspected publication bias. Recovery/fatigue - serious methodological limitations, very serious inconsistency, serious imprecision, no serious indirectness, and suspected publication bias. Publication bias was considered suspected because grey literature was not systematically searched, the search used outcome-constrained terminology, several studies were small or abstract-only, and selective-reporting concerns were common. All three domains therefore remain very low certainty.

## 4. Discussion

Across 45 reports representing 41 unique studies, the overall picture was mixed. Some trials reported favourable comparative findings for repeated-sprint performance, muscular strength, and anaerobic power, but many others reported null effects, within-group-only changes, or results that could not be interpreted confidently as between-group effects. Certainty was very low across all broad outcome domains. Vertical-jump findings were inconsistent, and no clear pattern of benefit emerged for change-of-direction ability or endurance. Technical-skill findings were also mixed: some studies reported favourable dribbling outcomes, but kicking accuracy did not improve consistently. Physiological and biochemical outcomes, including lactate, heart rate, muscle-damage markers, and inflammatory indices, were mostly null or inconsistent. Body mass commonly increased, especially with loading protocols, while evidence for increases in lean or muscle mass remained limited and inconsistent.

Compared with previous reviews, our findings are broadly consistent in identifying repeated-sprint performance, anaerobic power, and muscular strength as domains in which some favourable findings have been reported. However, the present review found that comparative effects were inconsistent and the certainty of evidence was very low, precluding firm conclusions regarding efficacy. Mielgo-Ayuso et al. similarly reported beneficial effects primarily for anaerobic performance and Wingate outcomes, with less consistent effects for aerobic performance [18]. Compared with earlier reviews, the present review included a substantially larger evidence base (45 reports representing 41 unique studies) and additionally evaluated body composition, recovery/fatigue-related outcomes, biochemical markers, and technical skills, providing a broader synthesis of the literature.

The more recent review by Monsalve Guevara et al. also summarised the effects of creatine in soccer players but included only a limited number of studies and primarily focused on performance-related variables [64]. In contrast, our review substantially expanded the available evidence base and demonstrated considerable heterogeneity across supplementation strategies, duration, participant characteristics, training status, and outcome assessments. Although the previous review suggested that a loading-plus-maintenance approach might produce more consistent ergogenic effects, the present review did not formally compare supplementation strategies and therefore does not establish the superiority of maintenance protocols. In addition, Luo et al. [6], in a network meta-analysis of dietary supplements in soccer players, reported small beneficial effects of creatine on jump and anaerobic performance outcomes [6]. Our findings partially align with these results, although vertical jump performance remained inconsistent across creatine-specific studies.

Soccer combines aerobic work with repeated bursts of high-intensity activity and requires players to perform technical actions while fatigued. Sprints, accelerations, decelerations, changes of direction, jumps, passing, shooting, and dribbling all place different demands on the player, which makes it unlikely that a single supplement will affect every aspect of performance in the same way.

Creatine nevertheless has a clear physiological rationale in this setting. By increasing intramuscular creatine and phosphocreatine availability, supplementation may support ATP resynthesis during repeated high-intensity efforts separated by short recovery periods. In principle, this could help players sustain repeated maximal or near-maximal actions and preserve technical execution as fatigue develops. The soccer-specific evidence, however, did not show a consistent benefit for dribbling or ball-speed outcomes, so this proposed link remains biologically plausible rather than demonstrated.

The same complexity may also explain why plausible physiological benefits did not translate consistently into soccer-specific performance. Short-term creatine protocols often increased body mass, but the magnitude and comparative significance of that gain varied. Additional body mass or muscle water could plausibly affect locomotor demands, yet this review did not test whether changes in body mass mediated sprint, endurance, or change-of-direction outcomes. Nor did the available evidence establish that loading is better than non-loading, that longer protocols are more effective than shorter ones, or that any particular dose is superior. These remain hypotheses informed by the wider creatine literature rather than conclusions from the soccer-specific evidence assembled here.

The ergogenic effects of creatine in soccer can be explained through several interrelated physiological mechanisms. The most established pathway is the augmentation of intramuscular phosphocreatine stores, which accelerates ATP resynthesis during high-intensity bouts and reduces reliance on anaerobic glycolysis [65]. This mechanism is relevant to repeated-sprint ability (RSA) and peak power output; however, the included soccer studies did not consistently demonstrate comparative benefits for these outcomes. Faster phosphocreatine resynthesis may reduce energetic strain during repeated efforts, but the review did not establish that supplementation enables players to maintain high-intensity output throughout an actual match.

The phosphocreatine-creatine kinase reaction can contribute to intracellular proton buffering during high-intensity exercise, providing a plausible link between creatine availability and acid-base regulation [66]. Broader physiological literature also supports a buffering role for the creatine kinase system [67]. However, the included soccer studies reported inconsistent changes in blood lactate, pH, and bicarbonate and did not establish intracellular buffering as the mechanism responsible for any performance effect.

For biochemical responses, individual trials reported smaller exercise-related changes in LDH or inflammatory markers, although findings were inconsistent and do not establish a protective effect against muscle damage or inflammation. Reductions in plasma homocysteine, observed in some studies, may suggest potential physiological relevance [68,69], although the clinical significance of these findings remains unclear, while the absence of significant effects on oxidative stress markers or antioxidant enzyme activity suggests limited influence on redox balance in this population.

## 5. Practical implications

The findings do not support firm soccer-specific recommendations about whether creatine works, when it should be taken, or what dose or protocol is optimal. Although some trials reported favourable effects on repeated-sprint performance, anaerobic power, and muscular strength, the comparative evidence was inconsistent and of very low certainty. Creatine should therefore not be presented as a reliably effective way to improve match performance, recovery during congested schedules, or position-specific performance.

If creatine is used in practice, the decision should be individualised to the player's training goals, diet, previous supplementation experience, tolerance, and the possible consequences of gaining body mass. Body mass increased frequently, particularly with loading protocols, but it remains unclear whether these changes help or hinder sprinting, endurance, change-of-direction performance, or position-specific demands. Monitoring body mass, gastrointestinal symptoms, and general tolerance is therefore reasonable.

The included studies also did not provide enough comparative evidence to identify a superior dose, loading or maintenance strategy, ingestion timing, or phase of the competitive season. Any specific dosing advice should therefore be drawn from broader creatine research or consensus guidance rather than from this soccer-specific review. Likewise, the current evidence does not show that creatine improves recovery between matches, preserves technical performance under fatigue, or benefits particular playing positions more than others.

For practitioners, creatine may be considered a potentially useful option for selected performance goals, but its soccer-specific effects are not firmly established. It is best trialled during training, with attention to body-mass changes and tolerability, and it should complement rather than replace established nutrition, recovery, and training practices. Better-powered, well-controlled trials are still needed before confident soccer-specific recommendations can be made about dose, timing, season phase, position, or recovery use.

## 6. Strengths and limitations

A key strength of this review is its exclusive focus on soccer and its inclusion of performance, technical, physiological, biochemical, and body-composition outcomes. By bringing together 45 reports representing 41 unique studies across different ages, competitive levels, and supplementation protocols, the review provides a broad picture of both the potential effects of creatine and the uncertainty that remains.

However, several limitations should be acknowledged. Considerable heterogeneity existed among the included studies in terms of participant characteristics (e.g. sex, age, and competitive level), supplementation protocols, creatine dosage, intervention durations, outcome measures, and performance testing procedures. Many trials involved small sample sizes, limiting statistical power and the generalisability of the findings. Dietary intake and training load, two major confounders, were rarely controlled or adequately reported, making it difficult to isolate the independent effects of creatine supplementation from other performance-related determinants. Inconsistencies in testing protocols (e.g. different sprint distances, varying Wingate parameters, or non-standardised technical skill tests) hindered direct comparison between studies. In addition, only a limited number of studies were conducted in female soccer players, making it difficult to draw sex-specific conclusions regarding the effects of creatine supplementation and highlighting the need for further research in female athletes. At the review level, the search was limited to three bibliographic databases and used outcome-related search terms, which may have reduced sensitivity for reports not indexed using the prespecified outcome terminology; grey literature and unpublished studies were not systematically searched. The final database search was conducted on 15 May 2025, so studies published subsequently may not be represented. Risk of bias was frequently identified across the included studies. Among randomised parallel-group trials, the principal concerns involved missing outcome data and selection of the reported result, whereas outcome measurement was generally judged at low risk. Among studies assessed with ROBINS-I, confounding was the most consistent source of serious risk of bias. In line with the GRADE approach, the certainty of evidence was rated as very low for body composition, performance, and recovery/fatigue-related outcomes, mainly due to methodological limitations, inconsistency across studies, suspected publication bias, and/or imprecision. The risk-of-bias figures summarise one methodological profile per study rather than formal result-specific assessments; outcome-specific bias may therefore differ within a study. The evidence base also mixes randomised and non-randomised designs, includes several abstract-only or incompletely reported studies, and could not be quantitatively weighted by effect size or precision. The domain-level GRADE judgements and narrative synthesis consequently remain broad summaries rather than outcome-specific pooled certainty estimates. Reference-list checking was used as a supplementary identification step, but yields from citation searching were not prospectively logged as a separate PRISMA identification stream. Furthermore, although broader literature has examined creatine as a potential nutritional strategy during injury recovery [70], no included study specifically evaluated creatine supplementation during rehabilitation from a soccer-related injury.

## 7. Conclusion

Overall, the evidence for creatine supplementation in soccer players is suggestive but inconsistent, and certainty across the broad outcome domains is very low. Some trials reported favourable findings for repeated-sprint performance, anaerobic power, and muscular strength, but the comparative evidence was heterogeneous and frequently limited by small samples, methodological concerns, or unclear and nonsignificant between-group findings. Firm conclusions about efficacy are therefore not warranted. Effects on vertical jump, change-of-direction ability, endurance, technical skills, recovery-related biomarkers, and inflammatory responses remain mixed or inconclusive. Body mass commonly increased, particularly after loading protocols, while convincing evidence for meaningful gains in lean mass was limited. Future studies should use adequately powered randomised designs, soccer-specific outcomes, transparent reporting, and systematic adverse-event monitoring.

## Supplementary Material

Supplementary Material Supplementary_Table_S1CleanVersion.docx
