# Supplementary material for: Creatine supplementation in soccer players: a systematic review
Source: J Int Soc Sports Nutr. 2026 Oct 2;23(Suppl 1):2735569. doi: 10.1080/15502783.2026.2735569 (PMC13644428; doi:10.1080/15502783.2026.2735569)
Supplement: Supplementary file 1 [file RSSN_A_2735569_SM9918.docx]

**Supplementary Table S1.** Full search strategies for each database

| **Database** | **Search Strategy** |
| --- | --- |
| **PubMed** | ("creatine" OR "creatine monohydrate" OR "creatine supplementation") AND ("soccer" OR "football" OR "football players" OR "soccer players") AND ("performance" OR "body composition" OR "fatigue" OR "recovery" OR "muscle strength" OR "sprint" OR "power" OR "jump" OR "agility") |
| **Scopus** | TITLE-ABS-KEY ("creatine" OR "creatine monohydrate" OR "creatine supplementation") AND TITLE-ABS-KEY ("soccer" OR "football" OR "football players" OR "soccer players") AND TITLE-ABS-KEY ("performance" OR "body composition" OR "fatigue" OR "recovery" OR "muscle strength" OR "sprint" OR "power" OR "jump" OR "agility") |
| **Web of Science** | TS = (("creatine" OR "creatine monohydrate" OR "creatine supplementation") AND ("soccer" OR "football" OR "football players" OR "soccer players") AND ("performance" OR "body composition" OR "fatigue" OR "recovery" OR "muscle strength" OR "sprint" OR "power" OR "jump" OR "agility")) |

**Abbreviations:** TITLE-ABS-KEY = title, abstract, and keyword fields; TS = topic search.

Search reporting note: all three databases were searched from inception through 15 May 2025. Records retrieved before deduplication were PubMed n=921, Web of Science n=719, and Scopus n=1,185. No language restriction was applied. The mandatory outcome-term block may have reduced sensitivity for reports indexed primarily under unlisted physiological/biochemical outcomes; this is acknowledged as a review limitation. Reference-list checking was supplementary, but its yields were not prospectively logged as a separate PRISMA identification stream.
